# LESSONS FROM THE PAST, VISIONS FOR TOMORROW: GERONTOLOGY AND GSA IN RETROSPECT

**DOI:** 10.1093/geroni/igae098.1649

**Published:** 2024-12-31

**Authors:** Soomi Lee, Soomi Lee

**Affiliations:** Chair; Discussant

## Abstract

GSA is approaching its 80th anniversary, prompting us to reflect on the origins and rich history of gerontology. Gerontology’s roots trace back to the early 1900s, an era where older age was often perceived solely as a period of physical and mental decline. However, over time, our understanding of aging has significantly evolved. Despite this progress, a noticeable gap persists in inter-generational knowledge exchange, hindering the opportunity to build upon previous theories and extend seminal work. This year’s theme, “fortitude,” underscores the importance of sustaining good science of aging across generations and disciplines. In response, our symposium provides an educational and scholarly exchange platform rooted in historical perspectives. Three esteemed panelists who have played pivotal roles in shaping GSA’s trajectory will share their insights and experiences, with each presentation followed by comments by other panelists. Dr. Steven H. Zarit, recipient of the 2019 Robert W. Kleemeier Award, will discuss how the field of gerontology has evolved. Dr. David Chiriboga, recipient of the 2010 Hiram J. Friedsam Outstanding Mentorship Award, will share his memorable mentorship stories. Dr. Kathleen Wilber, recipient of the 2023 Donald P. Kent Award, will address key values and challenges of the field. The moderator, Dr. Soomi Lee, will facilitate discussions with the panelists and audience. This session offers an opportunity for the Academy for Gerontology in Higher Education (AGHE) and the wider GSA community to reflect on the lasting impact of gerontology and discuss collective visions for tomorrow as we navigate the evolving landscape of the field. Generativity and Aging Interest Group Sponsored Symposium

